# Bio-Inspired Design of a Porous Resorbable Scaffold for Bone Reconstruction: A Preliminary Study

**DOI:** 10.3390/biomimetics6010018

**Published:** 2021-03-10

**Authors:** Daria Scerrato, Alberto Maria Bersani, Ivan Giorgio

**Affiliations:** 1 Dipartimento di Scienze di Base ed Applicate per l’Ingegneria (SBAI), University of Rome La Sapienza, 00161 Roma, Italy; daria.scerrato@uniroma1.it; 2 International Research Center for the Mathematics and Mechanics of Complex Systems (M&MoCS), University of L’Aquila, 67100 L’Aquila, Italy; alberto.bersani@sbai.uniroma1.it; 3 Dipartimento di Ingegneria Meccanica e Aerospaziale (DIMA), University of Rome La Sapienza, 00184 Roma, Italy; 4 Dipartimento di Ingegneria Civile, Edile-Architettura e Ambientale (DICEAA), University of L’Aquila, 67100 L’Aquila, Italy

**Keywords:** bone remodeling, bone/graft interaction, computer-aided design, metamaterials, second gradient materials

## Abstract

The study and imitation of the biological and mechanical systems present in nature and living beings always have been sources of inspiration for improving existent technologies and establishing new ones. Pursuing this line of thought, we consider an artificial graft typical in the bone reconstruction surgery with the same microstructure of the bone living tissue and examine the interaction between these two phases, namely bone and the graft material. Specifically, a visco-poroelastic second gradient model is adopted for the bone-graft composite system to describe it at a macroscopic level of observation. The second gradient formulation is employed to consider possibly size effects and as a macroscopic source of interstitial fluid flow, which is usually regarded as a key factor in bone remodeling. With the help of the proposed formulation and via a simple example, we show that the model can be used as a graft design tool. As a matter of fact, an optimization of the characteristics of the implant can be carried out by numerical investigations. In this paper, we observe that the size of the graft considerably influences the interaction between bone tissue and artificial bio-resorbable material and the possibility that the bone tissue might substitute more or less partially the foreign graft for better bone healing.

## 1. Introduction

Design of new materials with continuously increasing performance has always been a challenge for scientists and engineers. An approach rather promising is to study natural materials with non-common properties and try to reproduce their responses with new artificial solutions. In other words, the design of new synthetic materials is bio-inspired. For example, bone tissue, like other natural materials, is able to adapt its internal structure to the demands of the environment. Although some efforts to conceive a similar material have been made in recent past years, the way to cover for obtaining materials with the same features is still rather long [1,2]. The endeavor to conceive a material capable of mimicking the outstanding bone characteristic can be partially mitigated if one considers integrating the bone tissue with a synthetic graft. In this kind of ‘composite’ system, the primary objective is to design the graft in the best possible way to speed up the process of healing of an injured bone or, in the case of a disease that affects the tissue itself. On the one hand, some living bone tissue features will be inherited by the graft because of the system of cells that act already on that tissue and can, in some way, also perform on the graft material if this last is resorbable. On the other hand, it will be the inner microstructure of the graft that may accelerate or hinder the healing. Here, therefore, we focus on studying the interaction of the graft with the bone to provide guidelines for designing the structure of the implant. We already know that the graft must be porous and sufficiently strong to allow the bone remodeling and guarantee the mechanical strength required to carry on the entire process. However, in-depth knowledge of this kind of interaction is essential to improve the effectiveness of the graft to be designed. For all intents and purposes, that knowledge can be refined by employing suitable models of the system based on simplifying hypotheses that allow it to be described with sufficient accuracy and that will be corroborated experimentally. In the examined case, we have to deal not only with a complex mechanical system, such that the usual approach may be unsuccessful, but also with an evolution of the mechanical properties in the time due to the adaptation process taking place, and hence it is driven by mechanical and biological aspects. An efficient way to consider the typical complexity of the bone tissue, also with the addition of a graft intended to be as much as possible similar to the bone itself, is the use of generalized continuum theories (see, e.g., [3,4,5,6,7]) where the effects of the microstructure lying at the low levels of observation are introduced in the model as further kinematical descriptors that enhance the possibilities of the description of the system at the macro level. Together with poroelastic formulations, we believe that a second gradient theory should be considered because of the multi-scale organization of the bone tissue and its heterogeneity that involves the presence of high contrast in the mechanical properties of the constitutive elements of the microstructure [8,9,10]. This is a key factor to require the need of the second gradient continuum model. Besides, the consideration of the gradient of the deformation in the mechanical formulation is also beneficial for modeling the stimulus, which activates the adaptation process, [11,12,13,14] since it can be read as a macroscopic source of canalicular fluid flow that is commonly assumed to have a crucial role in the remodeling process [15,16].

To design the graft, a promising method is the one typically used to conceive metamaterials [17,18,19,20,21,22,23]. The opening gambit is the definition of the functional requirements of the graft, which are conveyed in a specific behavior to obtain. Then, the design effort is devoted to synthesizing the microstructure that can ensure the data specifications required. In this work, we assume that the graft has a microstructure very similar to the bone tissue; therefore, we model them, bone and graft, within the same formulation. However, we remark that the graft can be modeled with different mechanical properties to enhance its healing capability for future developments (see, e.g., [24,25,26,27,28]).

## 2. The Model Employed to Describe Bone Remodeling

### 2.1. Mechanical Formulation

The system under study is constituted by a trabecular bone tissue to which an artificial graft, with a similar microstructure, is added to facilitate the healing process following a dire injury or a fracture. The main aim of the graft is to provide a junction that restores the mechanical continuity of the broken bone and a scaffold that allows the bone-cells responsible for the remodeling process to operate in resorbing the extraneous bio-material and substituting it with freshly produced bone. Since in this paper, we examine a preliminary case to illustrate as the proposed model can be beneficial from a computational point of view, bone is treated in general terms. Namely, no distinctions are made concerning the particular kind of bone (i.e., long bones, flat bones, etc.). The only hypothesis we consider is that the bone is trabecular because, in this case, the size-effect that a second gradient model can describe could be more evident. We are well aware that the effects of mechanical usage and biochemical agents probably occur at different rates and in various proportions for diverse skeleton regions. However, the hypotheses beneath this formulation are pervasive for all kinds of bone. From a modeling viewpoint, both the bone and the artificial graft can be described by a porous material. This is quite natural in the bone case, while some remarks should be made for the graft. Indeed, the bone tissue is characterized by different kinds of porosity, significant at various scales of observations, namely inter-trabecular, vascular, lacunar-canalicular, and collagen-apatite porosity [29,30]. In the case of the graft, instead, we can design it to the best following a suitable criterion that depends on the particular case examined. In most clinic cases, the objective is to use the graft as temporary support for the cells involved in the remodeling process that, at the end of the healing, will result in a more or less complete substitution of the graft with healthy bone. This perspective demands a graft as a material with inner cavities, recommendably interconnected, to supply the remodeling cells with the room to be able to carry out the process to which they are predisposed as well as with their nutrients.

Therefore, the investigation of the considered system is performed by using a generalized continuum model endowed by an extra-kinematical descriptor that takes into account the deformability of the micro-structure, namely the change of porosity, observed at the macro-level. In this framework, we assume that the significant behavior of the deformable body is completely determined when are known:

1. the bulk displacement vector, u=x−X,
2. the Lagrangian porosity, ϕ,

where we used the standard definitions. Specifically, X represents any material particle of the body in the reference configuration ($B^{*}$), x=χ(X,t) is the place occupied by the particle X at the time *t* in the current configuration, and the Lagrangian porosity ϕ, assumed scalar, is the *current void density* referred to the reference total space, i.e., the current void space is evaluated as

(1) $$V_{void}=\int_{B^{*}}\phi \left(X\right)\;dB^{*}$$

Analogously, one can define the Eulerian porosity as

(2) $$V_{void}=\int_{B}n\left(x\right)\;dB$$

which is the corresponding current void density related to the current total space B. The two porosities, naturally are linked together by the relationship

(3) $$\phi \left(X,t\right)=n\left[\chi (X,t)\right]\;J\left(X,t\right)$$

being J=detF=det∇χ [31].

Therefore, with these kinematical descriptors the following deformation measures are allowed:

1. the *finite strain tensor*, E, whose components are
  (4) $$E_{ij}\left(X,t\right)=\frac{1}{2}\left(u_{i,j}+u_{j,i}+u_{i,k}\;u_{k,j}\right)$$
2. the *change of the Lagrangian porosity* [32]
  (5) $$\zeta \left(X,t\right)=\phi \left(X,t\right)-\phi^{*}\left(X,t\right)$$
  ϕ and $\phi^{*}$ being the Lagrangian porosity corresponding to the current and the reference configuration, respectively. We can also express ζ by the equation
  (6) ζ=−∇·[φ(U−u)]=−∇·w
  where U is the average fluid-displacement vector defined in such a way that the volume of fluid displaced through unit areas is φU and w=φ(U−u) represents the flow of the fluid relative to the solid but measured in terms of volume per unit area of the bulk medium.

Herein, we assume the finite strain tensor because of the microstructure that in some circumstances brings to a non-linear response nonetheless in small displacement and deformation regime as, e.g., it is documented in [33].

The considered system made of two parts, the bone tissue and the bio-resorbable graft, can be studied as a whole exploiting the mixture theory. Indeed, this theory has been introduced to describe a multiphase system made of several interpenetrable constituents. In other words, at any instant of time, all phases can be present at each material point. In particular, the bone tissue mainly has a solid phase and a porous space that is typically filled with bone marrow, interstitial fluids, blood, bone cells, etc. (see for more details [34]). The graft also is a porous material and when it is first placed onto the injured bone typically has empty pores. Those, soon enough, are filled with organic matter, like for the bone, in the healing process [35,36,37,38]. Therefore, after the first healing stage, the two parts are more or less in the same conditions. It is at this stage that we start to analyze the system. Clearly, even though the two considered parts start from a very similar initial configuration, their evolution due to the remodeling process is quite different. As a matter of fact, within the bone tissue, only a change of apparent mass density (the label apparent is used because the mass density is evaluated concerning the apparent or external volume of the body, including its pore volume) can be experienced. In contrast, in the bio-resorbable graft, together with the artificial material resorption (by osteoclasts), bone cells (osteoblasts) may synthesize new bone tissue. During the evolution of the remodeling process, inside the artificial graft, may coexist primarily three phases: bioresorbable material, bone, and physiological fluid insides pores.

In accordance with the mixture theory, the reference porosity can be expressed as follows

(7) $$\phi^{*}\left(X,t\right)=1-\left(\frac{\rho_{b}^{*}\left(X,t\right)}{{\hat{\rho }}_{b}}+\frac{\rho_{m}^{*}\left(X,t\right)}{{\hat{\rho }}_{m}}\right)$$

depending on the volume fractions of the constituents, namely $\rho_{b}^{*}/{\hat{\rho }}_{b}$ and $\rho_{m}^{*}/{\hat{\rho }}_{m}$, where $\rho_{b}^{*}$ and $\rho_{m}^{*}$ are the apparent mass densities of bone tissue and artificial material in the reference configuration, respectively; while the superimposed hat denotes the mass densities evaluated considering the true volume occupied by those phases. We remark that the apparent mass densities of the different phases evolve with time depending on the remodeling process and the mechanic environmental loads. Therefore, we assume the reference configuration of the continuum system is related to the apparent mass density in the reference state, i.e., when zero stress occurs. Eventually, the current porosity can be evaluated by

(8) $$\phi =\zeta +\phi^{*}$$

A possible choice for the elastic energy density coherent with the assumptions made and frame-indifferent is

(9) $$E=\tilde{E}\left(E,\nabla E,\zeta ,\nabla \zeta \right)$$

The strain gradient tensor is adopted to model size and non-local effects observed in the considered continuum system [39,40,41,42,43,44]. The presence of ζ in the list of meaningful variables follows the suggestion by Biot [32] and Cowin [45] to model porous materials. The dependency on ∇ζ allows us to deal with boundary conditions related to the change of porosity. In classical poromechanics, this effect is usually neglected. Still, we believe that to model the interaction at the interface between bone and graft in detail, these kinds of boundary conditions should be taken into account. Indeed, a term in the energy involving ∇ζ permits the usage of the pore pressure at the bone/graft interface as a boundary condition between the two parts. In particular, we consider the following energy density [46,47,48]:

(10) $$\begin{array}{c} E=\frac{1}{2}\lambda \left(\rho_{b}^{*},\rho_{m}^{*}\right)\;E_{\mathrm{ii}}E_{\mathrm{jj}}+\mu \left(\rho_{b}^{*},\rho_{m}^{*}\right)\;E_{\mathrm{ij}}E_{\mathrm{ij}}+\\ 4\;\alpha_{1}\left(\rho_{b}^{*},\rho_{m}^{*}\right)E_{\mathrm{ii},j}E_{\mathrm{jk},k}+\alpha_{2}\left(\rho_{b}^{*},\rho_{m}^{*}\right)E_{\mathrm{ii},j}E_{\mathrm{kk},j}+4\;\alpha_{3}\left(\rho_{b}^{*},\rho_{m}^{*}\right)E_{\mathrm{ij},i}E_{\mathrm{kj},k}+\\ 2\;\alpha_{4}\left(\rho_{b}^{*},\rho_{m}^{*}\right)E_{\mathrm{ij},k}E_{\mathrm{ij},k}+4\;\alpha_{5}\left(\rho_{b}^{*},\rho_{m}^{*}\right)E_{\mathrm{ij},k}E_{\mathrm{ik},j}+\\ \frac{1}{2}K_{1}\left(\rho_{b}^{*},\rho_{m}^{*}\right)\zeta^{2}+\frac{1}{2}K_{2}\zeta_{,i}\zeta_{,i}-K_{3}\left(\rho_{b}^{*},\rho_{m}^{*}\right)\;\zeta \;E_{\mathrm{ii}} \end{array}$$

where all the material parameters are considered a function of the apparent mass density of the bone and the graft evaluated in the reference configuration. Particularly, the coefficients $\alpha_{i}$ are the second gradient rigidities and $K_{j}$ are the stiffnesses due to the porous nature of the system. Naturally, since they evolve with the remodeling process also the behavior of the considered system will change accordingly during the time. This is the simplest choice that one can conceive due to the isotropic assumption. Albeit, we are well aware that more complex models can be considered to describe the anisotropic nature of the bone [49,50], we refrain from considering this kind of complexity essentially because well-established remodeling process formulations are not yet available for these cases. Besides, the approximation due to the isotropic behavior, even though simplistic, in many real clinic instances, can be acceptable [51]. By introducing the Young modulus of the mixture as a power law of the solid-phases volume fractions

(11) $$Y=Y_{b}^{\mathrm{Max}}{\left(\frac{\rho_{b}^{*}}{{\hat{\rho }}_{b}}\right)}^{2}+Y_{m}^{\mathrm{Max}}{\left(\frac{\rho_{m}^{*}}{{\hat{\rho }}_{m}}\right)}^{2}$$

$Y_{b}^{\mathrm{Max}}$ and $Y_{m}^{\mathrm{Max}}$ being the maximal elastic moduli, the standard Lamé parameters can be expressed in terms of the apparent mass densities as

(12) $$\lambda =\frac{\nu \;Y(\rho_{b}^{*},\rho_{m}^{*})}{\left(1+\nu )(1-2\nu \right)},\;\mu =\frac{Y(\rho_{b}^{*},\rho_{m}^{*})}{2(1+\nu )},$$

assuming constant the Poisson ratio for the sake of simplicity. The material parameters related to the second gradient term of the energy density are estimated as follows

(13) $$\begin{array}{c} \alpha_{1}=\alpha_{2}=\alpha_{4}=Y\left(\rho_{b}^{*},\rho_{m}^{*}\right)\ell^{2},\;\alpha_{3}=2Y\left(\rho_{b}^{*},\rho_{m}^{*}\right)\ell^{2},\\ \alpha_{5}=\frac{1}{2}Y\left(\rho_{b}^{*},\rho_{m}^{*}\right)\ell^{2} \end{array}$$

Herein, the role of the characteristic length *ℓ* is of paramount importance to consider the effect of boundary layers due to the microstructure. The order of magnitude of *ℓ* is assumed to be 200 μm, i.e., the diameter of trabeculae that are the elements of which the microstructure is made of (see, for more details on lattice structures similar to the trabecular bone, [10]). The second gradient continuum model may be deduced using an homogenization technique that through a micro-macro identification permit to obtain an equivalent model that predicts at the macro scale the response of the material due to the microstructure existing at micro-scale (see e.g., [26,52,53,54,55,56,57]). In this context, some critical issues related to the rise of damage in the system constituted by bone tissue and bio-material can be taken into account (see e.g., [58,59,60,61,62,63,64]).

The other three material coefficients $K_{1}$, $K_{2}$, and $K_{3}$ concern the presence of the pores and how they affect the macroscopic deformation equilibrium shapes of the system under study. Specifically, $K_{1}$ is a compressibility coefficient, namely the reciprocal of the poroelastic storage coefficient $S_{p}$, which is defined as the volume of fluid released from unit bulk volume per unit decrease in pore pressure under the condition of constant confining stresses [32], and it can be written as:

(14) $$K_{1}=\frac{1}{S_{p}}={\left(\frac{\phi^{*}}{K_{f}}+\frac{\left(\alpha_{B}-\phi^{*}\right)\left(1-\alpha_{B}\right)}{K_{\mathrm{dr}}}\right)}^{-1}$$

where $K_{f}$ is the stiffness of the fluid inside the pores and

$$K_{\mathrm{dr}}=\frac{Y}{3\;(1-2\nu )}$$

is the drained bulk modulus of the porous matrix, while $\alpha_{B}$ (being $\phi^{*}\leq \alpha_{B}\leq 1$) is the Biot-Willis coefficient. $K_{2}$ represents the aptitude of the system to oppose to the establishment of a gradient of porosity and, for the sake of simplicity, is assumed constant. Finally, $K_{3}$, as defined in [32], is expressed in the form

(15) $$K_{3}=\alpha_{B}\;K_{1}$$

being the coupling between the microstructure due to pores and the bulk solid. In the numerical simulations, we use a linear function of the porosity as

(16) $$\alpha_{B}=a_{1}\varphi^{*}+\left(1-a_{1}\right)$$

with $a_{1}=0.8$.

Albeit in the examined system, the sources of dissipation are many [65], foremost among those, we recall the dissipation: (1) in the bone solid matrix [66]; (2) in the fluid that fills the pores [67]; (3) at the interface between the solid and the fluid phase [68]; (4) at the interface between bone and graft [69]; (5) due to friction in possible micro-cracks within the solid phase [70]. Herein, we choose to treat all these sources from a macroscopic point of view, introducing a Rayleigh functional depending on the solid-matrix rate of deformation $\dot{E}$ as

(17) $$2D_{s}=2\mu^{v}\;\left({\dot{E}}_{\mathrm{ij}}{\dot{E}}_{\mathrm{ij}}-\frac{1}{3}{\dot{E}}_{\mathrm{ii}}{\dot{E}}_{\mathrm{jj}}\right)+\kappa^{v}\;{\dot{E}}_{\mathrm{ii}}{\dot{E}}_{\mathrm{jj}}$$

employing a Kelvin–Voigt model. In particular, in this formulation, the two contributions to the viscous dissipation, namely induced by a rate of shear deformation and a rate of hydrostatic deformation, are highlighted and particularized by the corresponding viscous coefficients $\mu^{v}$ and $\kappa^{v}$, respectively.

It is worth noting that several time scales characterize the considered system. As a matter of fact, there are at least two characteristic times: the one specified by the application of external mechanical loads, which is about a few seconds (for example, in walking, the average frequency can be estimated below 2.0 Hz [71]); the cyclic period due to the remodeling process (for cortical bone it is about 120 days, while for trabecular bone it is about 200 days [72]). Naturally, the system is more complicated. One can also add the characteristic time due to the evolution of the stimulus that, once released, will remain in the bone tissue for some hours and then reabsorbed by metabolic processes. Therefore, a detailed formulation of the problem could be developed with a multiple time-scale technique, but the discussion of this approach is beyond the purview of this paper. Herein, we decide to consider only the slow time scale related to the remodeling process. The fast dynamic due to the mechanical loads is considered only for the effects induced at the slow time scale. To be more precise, the inertial effects are neglected, while a more thorough explanation of the other aspects is needed. Thus, to determine the mechanical load at the considered time scale, the root mean square (RMS) of the external force is considered. The RMS represents the value (say the amplitude) of a time-constant or slowly variable force that dissipates the same power of the original force, which is time-varying. The key idea is to consider the force at the slow time scale as it is commonly done in electrical engineering, defining the RMS current value equivalent to a direct current, which dissipates the same power in a resistor. In other words, we consider that the external forces have two time characteristic period, one fast and one slow: (1) the former is addressed attributing to the amplitude of the force its RMS value; (2) the latter is considered with a time dependence of the force at the slow time-scale. It is worth noting that the RMS evaluation is performed on a time interval equal to the period of the considered quantity if this is a sinusoidal or any periodic function. Otherwise, the time windowing of the RMS integration should be calibrated based on the significant frequency content of the signal. Namely, it should include a sufficiently high number of cycles of the smallest frequency of the interest. Similarly, in the Equation (17), the velocity amplitude should be considered as an RMS value, therefore the dissipation is taken into account for the fast scale resorting to the RMS operator while for the slow scale with the Rayleigh functional itself.

The mechanical model of the system is therefore obtained by applying the Generalized Principle of Virtual Work in the following form

(18) $$\int_{B^{*}}\delta E\;dB^{*}+\int_{B^{*}}\frac{\partial D_{s}}{\partial {\dot{E}}_{\mathrm{ij}}}\delta E_{\mathrm{ij}}\;dB^{*}=\int_{B^{*}}\delta W^{\mathrm{ext}}\;dB^{*}$$

where $\delta W^{\mathrm{ext}}$ is the virtual work done by external loads. Compatibly with the postulated energy density (10), we are allowed to consider these possible external actions:

(19) $$\delta W^{\mathrm{ext}}=\int_{\partial_{\tau }B^{*}}\tau_{i}\delta u_{i}\;dS^{*}+\int_{\partial_{T}B^{*}}T_{\alpha }\delta u_{\alpha ,j}n_{j}\;dS^{*}+\int_{\partial_{\Xi }B^{*}}\Xi \;\delta \zeta \;dS^{*},$$

where the first term takes into account the forces per unit surface, i.e., $\tau_{i}$, on the part of the boundary $\partial_{\tau }B^{*}$, the second term is related to external double forces per unit of area [73], $T_{\alpha }$, which act on the normal part of the gradient of the displacement on the part of the boundary $\partial_{T}B^{*}$ [47,74], and the last term consider the effect of a pore pressure, Ξ, on the boundary $\partial_{\Xi }B^{*}$. The external virtual works done by body forces per unit volume, like the weight, and forces per unit line on boundary edges are neglected.

To model the interactions between the bone tissue and the artificial bio-resorbable graft, an extra-contribution of the virtual work (18) is defined by integrating upon the interface surface the following potential

(20) $$E_{\mathrm{int}}=\frac{1}{2}K_{u}\;\left[\;\left[u\right]\;\right]\cdot \left[\;\left[u\right]\;\right]+\frac{1}{2}K_{\nabla u}\;\left[\;\left[\left(\nabla u\right)n\right]\;\right]\cdot \left[\;\left[\left(\nabla u\right)n\right]\;\right]+\frac{1}{2}K_{\zeta }\;{\left[\;\left[\zeta \right]\;\right]}^{2}$$

where the symbol [[·]] represents the jump of a field f(X) from side to side through the interface, i.e., $\left[\;\left[f\right]\;\right]=\left(f^{+}-f^{-}\right)$. This potential describes in particular an elastic junction between the two phases, bone and graft, that is established for the kinematical fields u, ∇u, and ζ. The material rigidities $K_{u}$ and $K_{\nabla u}$, and $K_{\zeta }$ denote the effectiveness of the bond.

### 2.2. Growth/Resorption Process Formulation

This section details the model of the functional adaptation of the bone tissue with the inclusion of the bio-resorbable graft plugged in through surgery. Notably, we assume to describe this biological phenomenon by postulating the evolutive laws for the apparent mass density of the bone and graft in the reference configuration, namely when the stress within the system is zero. The simplest way to conceive these evolutions rules is by setting the time derivative of the mass densities as functions of a mechanical stimulus S(X,t) resulting from an external mechanical action and the current porosity ϕ(X,t). Typically, they are expressed as [35,75]:

(21) $$\begin{array}{c} \left\{\begin{array}{cc} \frac{\partial \rho_{b}^{*}}{\partial t}\left(X,t\right)=A_{b}\left(S\right)\;H\left(\phi \right) & \;\mathrm{with}\;0<\rho_{b}^{*}⩽{\hat{\rho }}_{b}\\ \frac{\partial \rho_{m}^{*}}{\partial t}\left(X,t\right)=A_{m}\left(S\right)\;H\left(\phi \right) & \;\mathrm{with}\;0<\rho_{m}^{*}⩽\rho_{m}^{*}\left(X,0\right) \end{array}\right. \end{array}$$

The functions $A_{b}$ and $A_{m}$ are assumed to be piece-wise linear:

(22) $$A_{\left\{b,m\right\}}\left(S\right)=\left\{\begin{array}{c} s_{\left\{b,m\right\}}S\;\mathrm{for}\;\;S\geq 0\\ r_{\left\{b,m\right\}}S\;\mathrm{for}\;\;S<0 \end{array}\right.$$

where the symbol $\left\{b,m\right\}$ stands for the alternative between b, i.e., bone, and m, i.e., material of graft. The coefficients *s* and *r* are the rates of growth and resorption, respectively. Clearly, the artificial material of the graft cannot be synthesized by the bone cells (osteoblasts); hence the coefficient $s_{m}$ must be zero.

The objective of the function *H* is to provide a tool for the calibration of limits of the apparent mass densities, namely a kind of end-of-stroke. In this context, the role of the porosity is vital. The pores allow the bone cells to reach the more remote zones of the system and operate to remove or synthesize bone tissue as demanded by the environmental external loads. Therefore, on the one hand, if the porosity is too low there is no room for this settlement, and the mass densities remain unchanged. On the other hand, if there is no physical support for the cells, i.e., high porosity, equally the remodeling cannot have a place. For these reasons, we can use a ∩-like shape function with its maximum approximatively near ϕ=0.5 where the most effective conditions in the remodeling process can be supposed (see for more details [65]).

The stimulus *S* is the key variable to model the process of functional adaptation. It acts as a feedback correction to the mechanical properties of the system. In other words, the bone system is capable to monitoring its mechanical state and integrity. It is commonly accepted that osteocytes are supposed to perform this measuring. This information is used to compare the current condition with a reference state and a possible deviation between these two states triggers a physiological activity of the bone cells to restore the lost equilibrium. Herein, we choose that the osteoblasts and osteoclasts change the apparent mass densities of the solid phases and, in turns, the mechanical rigidities characteristics of the system.

The acquired information of the mechanical state by the osteocytes represents a detailed picture of the bone/graft system. From a modeling viewpoint, this knowledge can be expressed in the integral form [65,76]:

(23) $$P\left(X,t\right)=\frac{\int_{\;B}\left[a_{1}\;E_{s}\left(X_{0},t\right)\;+a_{2}\;\;\int_{0}^{t}D_{s}\left(X_{0},\tau \right)d\tau \right]\;\varpi \left[\rho_{b}^{*}\left(X_{0},t\right)\right]\;e^{-\frac{∥X-X_{0}{∥}^{2}}{2D^{2}}}dX_{0}}{\int_{\;B}e^{-\frac{∥X-X_{0}{∥}^{2}}{2D^{2}}}dX_{0}}$$

which is a weighted average of the ‘mechanical state’ of the system. The exponential function defines the influence range of the osteocytes disseminated within the bone tissue, indeed. The radius of this area of influence is *D*. The farther the distance between the position of the osteocytes $X_{0}$ and the evaluation point of the signal X that will drive the osteoblasts and osteoclasts, the less powerful the signal level will be. The function ϖ denotes the density of osteocytes and acts as a gain for the ‘acquired’ mechanical state. Unquestionably, the more osteocytes there are, the better is for reconstruct the signal. Since the space density of the osteocytes is almost uniform in the bone tissue is reasonable to assume that ϖ is proportional to the bone density. In the numerical simulations, we set $\varpi =0.2\;\rho_{b}^{*}/{\hat{\rho }}_{b}$.

In Equation (23), the mechanical state is computed as a linear combination, whose coefficients are denoted by $a_{i}$, of the strain energy density $E_{s}$ of the solid matrix—including the first and second gradient displacement terms of Equation (10)—and the dissipated energy evaluated as the time integral of the dissipated power $D_{s}$. This particular option is dictated by the main mechanical objectives of the bone system, namely to provide weight-bearing capabilities, leverages for motion, and protection for the vital organs. Therefore, static strength can be assured looking to stored energy as a global figure of merit, while dynamic characteristics as shock-absorbing and vibration reduction could be achieved taking into account dissipative phenomena that can be linked to the rate of deformation. From a control point of view, this is very similar to a common proportional-derivative (PD) strategy (see, e.g., [77,78,79]).

We remark that the second gradient term in the present formulation is not only important for mechanical purposes but also to better describe the mechanism of functional adaptation. On the one hand, without this contribution we loose the possibilities of describes possibly boundary layers in the deformed shapes of the bone/graft system and size effects (the importance of these aspects is related to the characteristic length *ℓ*); on the other hand, the strain gradient can be considered as a macroscopic source of canalicular fluid flow that seems to have a strong correlation to the formation of bone in specific sites (see, e.g., [15,16,80,81,82]).

Finally, the stimulus can be written as

(24) $$S\left(X,t\right)=\left\{\begin{array}{cc} P\left(X,t\right)-P_{{}_{\mathrm{ref}}}^{s}\; & \mathrm{for}\;\;P\left(X,t\right)>P_{{}_{\mathrm{ref}}}^{s}\\ 0 & \mathrm{for}\;\;P_{{}_{\mathrm{ref}}}^{r}⩽P\left(X,t\right)⩽P_{{}_{\mathrm{ref}}}^{s}\\ P\left(X,t\right)-P_{{}_{\mathrm{ref}}}^{r} & \mathrm{for}\;\;P\left(X,t\right)<P_{{}_{\mathrm{ref}}}^{r} \end{array}\right.$$

where the two thresholds $P_{{}_{\mathrm{ref}}}^{s}$ and $P_{{}_{\mathrm{ref}}}^{r}$ delimitate the width of a lazy zone, i.e., an interval where the osteoregulatory balance of the bone tissue is preserved.

## 3. An Illustrative Theoretical Case: Numerical Implementation

**Figure 1:**
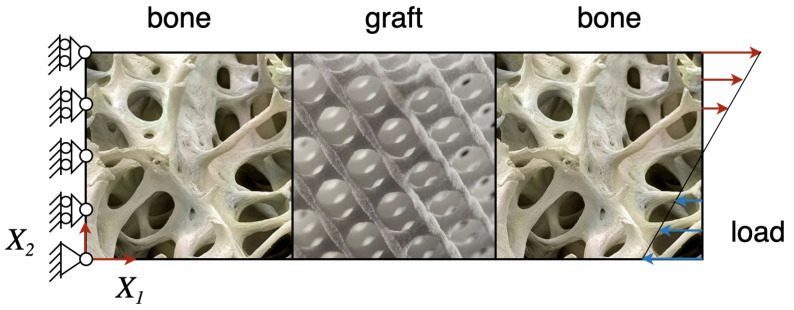
Schematic representation of the system under study. It is constituted by two pieces of bone tissues joined by a graft made of a bio-resorbable material, that is the BGB junction.

Although the proposed formulation is developed for the more general three-dimensional case, in the next sections, we consider a representative bi-dimensional example to better understand the potentialities of the proposed approach. Specifically, since the graft size typically varies from a few millimeters to some centimeters, we take into account a rectangular sample of size: 2×0.5 cm. We intend to study the interaction between the bone living tissue with a junction made with an artificial material that can be resorbed by the bone cells. This study aims to obtain beneficial criteria in the design of synthetic grafts to be used in bone reconstruction surgery. The sample comprises three sectors, two of bone tissue and a central one representing the graft, let say BGB junction for short. One side is constrained in order to avoid longitudinal displacement and any rigid motion keeping free the transverse displacement (see Figure 1). On the opposite side, a force per unit line with a symmetric linear distribution along the transverse direction is applied, as shown in Figure 1. The mechanical load is harmonically variable with a low frequency Ω, which directly affects the system at the considered time scale. We remind that the effects due to the fast oscillation of the force are incorporated in the model interpreting the magnitude of the force as an RMS value. In particular, we consider the longitudinal component of the force as follows

(25) $$f_{1}\left(X_{2},t\right)=\left(\frac{X_{2}}{w}-\frac{1}{2}\right)\left[F_{0}+F_{1}\sin\left(\Omega t\right)\right]$$

where *w* is the width of the specimen, the other amplitudes are set as: $F_{0}=1.68\times 10^{-3}\;Y_{b}^{\mathrm{Max}}$, $F_{1}=F_{0}/2$ and $\Omega =8.27\times 10^{-6}$ Hz, which corresponds to five cycles per week. The transverse force component is assumed zero.

The material parameters used in the simulations are summarized in Table 1.

The numerical simulations have been performed through a finite element analysis using the COMSOL Multiphysics software which is able to solve the coupling problem of Equations (18) and (21). The discretization mesh size is set after a ‘convergence’ analysis to provide an adequate degree of accuracy. In particular, when the total energy of the sample remains almost unchanged, varying the size of the mesh, we accept that value, namely D/4. We also remark that a particular numerical technique has been used to properly consider the second gradient terms of the energy in the model. As commonly done for the beam formulation, a micromorphic approach based on the use of Lagrange multipliers allow us to treat the second gradient model in the same framework as the standard first gradient model [83]. Alternatively, one can implement an ad hoc numerical formulation that guarantees an inherent high continuity for the unknown fields, namely the isogeometric analysis (see, e.g., [84,85,86,87,88,89,90]). A novel numerical algorithm has recently been developed based on a particle system that has shown a beneficial capability to model second gradient materials and multi-crack evolution [91,92].

## 4. Results and Discussion

In order to illustrate one of the foremost parameters involved in the remodeling process and, therefore, the actual predictive capability of the proposed modeling tool to be used in the design of the graft, we carried out some numerical simulations. In what follows, we study the influence of the graft size inside the BGB junction, keeping the other parameters unchanged. In particular, we choose the length of graft: 0.8, 0.6, 0.4, and 0.2 cm. They correspond to the 40%, 30%, 20%, and 10% of the entire sample length.

The initial configuration is characterized by a uniform distribution of apparent mass density in the bone sides corresponding to a volume fraction of the bone tissue $\rho_{b}^{*}/{\hat{\rho }}_{b}=0.5$; in the same way a uniform distribution of volume fraction for the graft material $\rho_{m}^{*}/{\hat{\rho }}_{m}=0.5$ is fixed.

Then, for the action of an external force, we promote bone growth in the BGB junction to analyze the effects of this mechanical stimulation by varying the size of the graft. The span of the time period analyzed is about 15 weeks to appreciate the evolution of the remodeling process.

**Figure 2:**
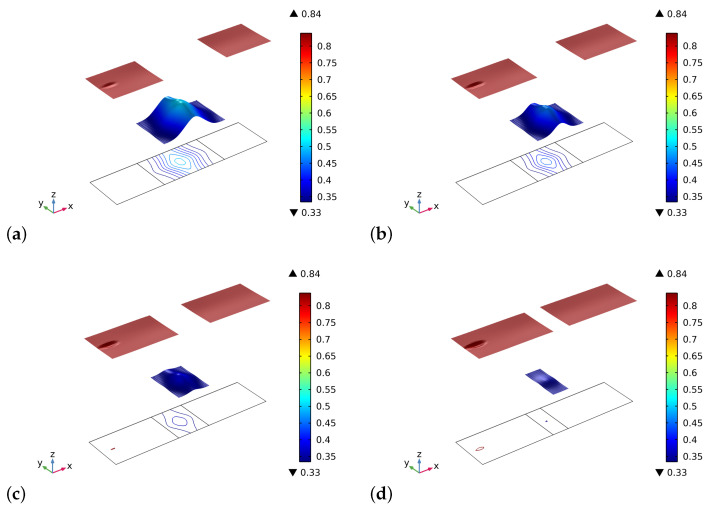
Plots of volume fraction of the bone tissue $\rho_{b}^{*}/{\hat{\rho }}_{b}$ at the end of the considered period for different substitute lengths of the graft: (**a**) 0.8 cm. (**b**) 0.6 cm. (**c**) 0.4 cm. (**d**) 0.2 cm.

**Figure 3:**
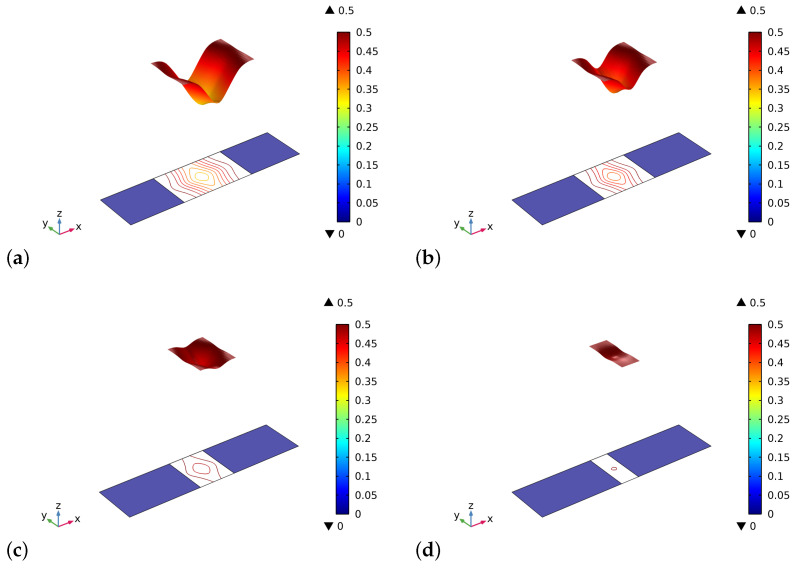
Plots of volume fraction of the graft material $\rho_{m}^{*}/{\hat{\rho }}_{m}$ at the end of the considered period for different substitute lengths of the graft: (**a**) 0.8 cm. (**b**) 0.6 cm. (**c**) 0.4 cm. (**d**) 0.2 cm.

Figure 2 and Figure 3 show the volume fractions of the bone and the graft material, respectively, at the end of the time interval of the simulations. The general trend we observe is that after an initial stage in which the artificial material is resorbed, at a different rate for the various cases in the graft part, the bone tissue starting from both lateral sides has also been synthesized in the central zone. Therefore, in the bone sectors, the volume fraction reaches saturation of the produced bone, and in the central zone, the two phases of bone and bio-resorbable material coexist at the same time.

**Figure 4:**
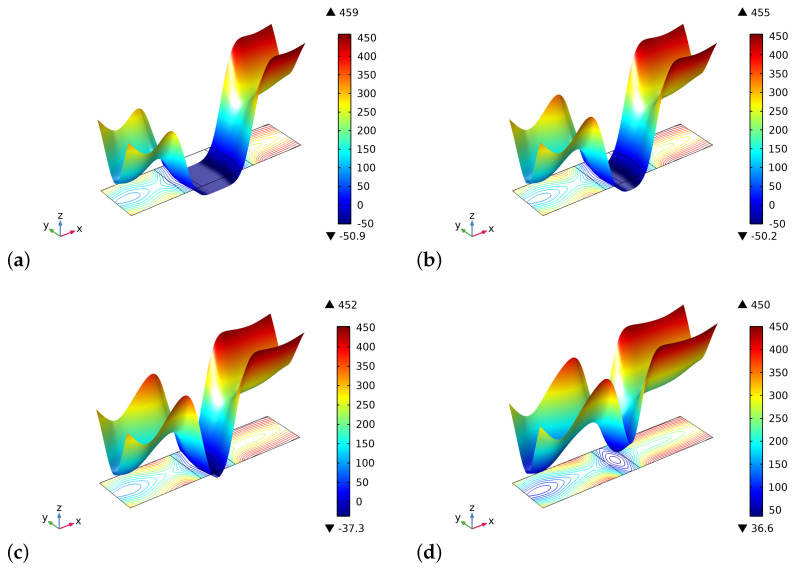
The initial distribution of the stimulus *S* as determined by the external actions for different substitute lengths of the graft: (**a**) 0.8 cm. (**b**) 0.6 cm. (**c**) 0.4 cm. (**d**) 0.2 cm.

The different results in the behavior are a direct consequence of the initial distribution of the stimulus (see, Figure 4). Indeed, in the graft at the beginning of the process, there are not osteocytes, and hence the level of the stimulus may be negative depending on the size of the graft. In the cases where the length of the graft is smaller, the stimulus can be positive because it is a collective signal from all the osteocytes present in a certain space zone. Thus the osteocytes near the graft in the bone living tissue can produce a strong enough stimulus to be positive even in the graft zone. Naturally, if the graft is larger, the influence of the neighborhood osteocytes at the center of the graft fades.

**Figure 5:**
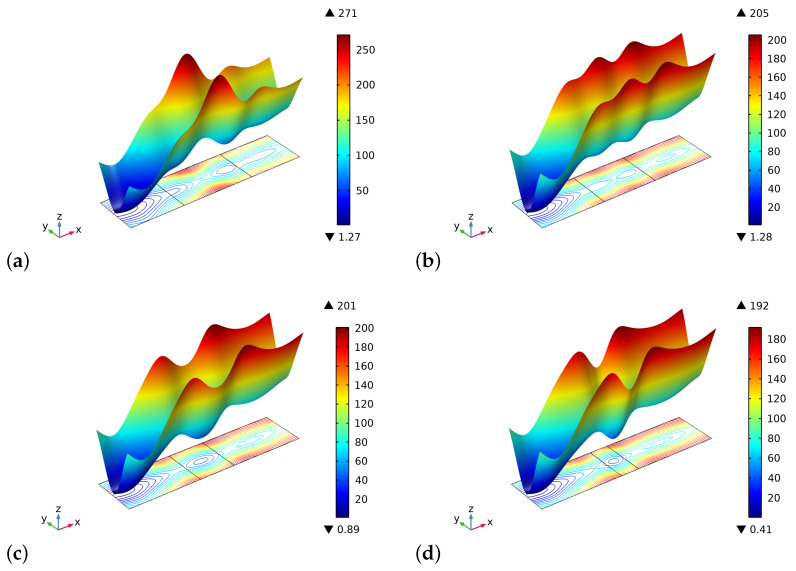
The distribution of the stimulus *S* at the end of the considered period for different substitute lengths of the graft: (**a**) 0.8 cm. (**b**) 0.6 cm. (**c**) 0.4 cm. (**d**) 0.2 cm.

At the end of the considered period, instead, the distribution of the stimulus is almost the same for the four cases examined because the entire sample reaches a state of saturation, and the small differences that can be detected are related to the different amount of bio-resorbable material which remains in the central zone (see, Figure 5). We notice herein that the artificial material and the bone tissue have a maximum (i.e., without pores) Young modulus slightly different. Besides, in the central zone where new bone tissue is also synthesized, new osteocytes are formed, and the stimulus level increases. At the end of the time interval of the simulation, notwithstanding the stimulus is positive, the remodeling process stops because the conditions for it are not any more favorable, i.e., the porosity is excessively small.

**Figure 6:**
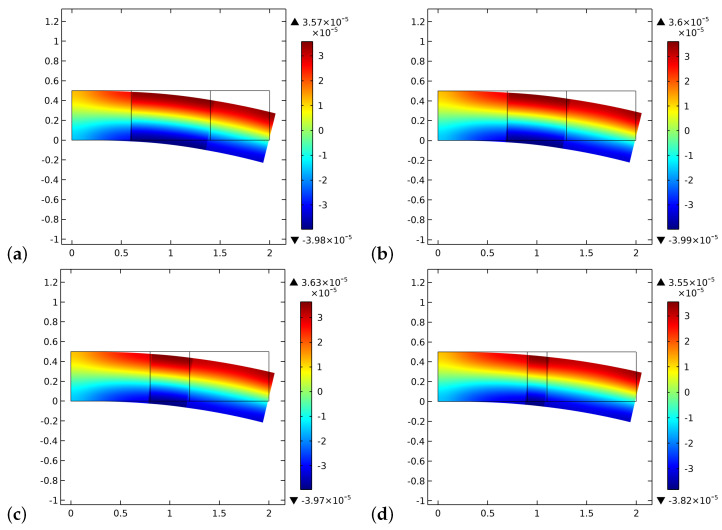
The distribution of ζ at the beginning of the time of simulation for different substitute lengths of the graft: (**a**) 0.8 cm. (**b**) 0.6 cm. (**c**) 0.4 cm. (**d**) 0.2 cm.

**Figure 7:**
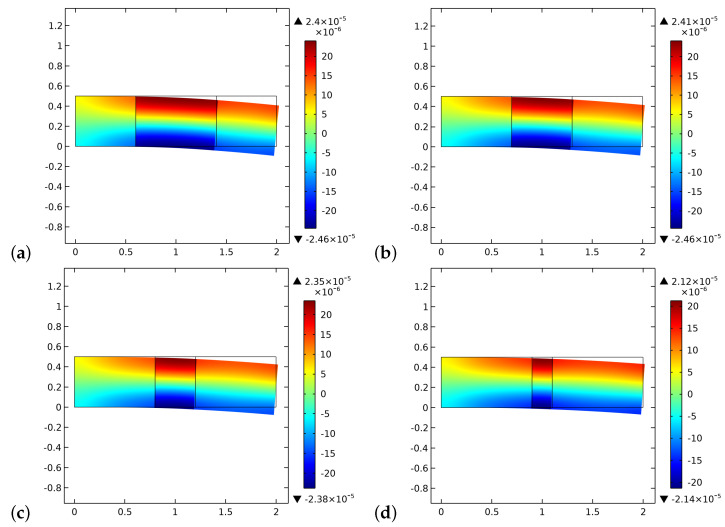
The distribution of ζ at the end of the time of simulation for different substitute lengths of the graft: (**a**) 0.8 cm. (**b**) 0.6 cm. (**c**) 0.4 cm. (**d**) 0.2 cm.

Figure 6 and Figure 7 display the initial and final distributions of the change of porosity ζ. From these pictures, it is clear that the process results in a stiffer system with a low level of porosity. Besides, we notice the differences due to the presence of the graft, especially at the bone-graft interface. The equilibrium shapes are plotted here and for the next figures, not in actual scale but with the same magnification (20×) to better recognize the kind of deformation.

**Figure 8:**
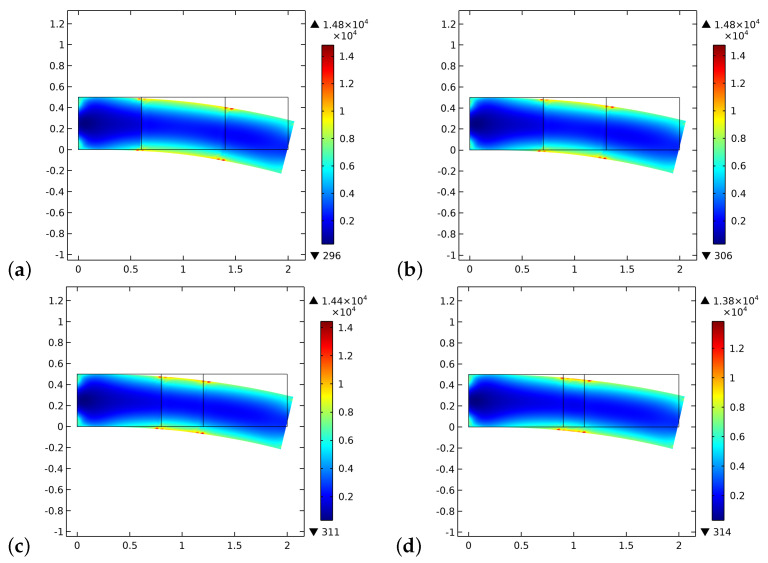
The distribution of the energy density E at the beginning of the time of simulation for different substitute lengths of the graft: (**a**) 0.8 cm. (**b**) 0.6 cm. (**c**) 0.4 cm. (**d**) 0.2 cm.

**Figure 9:**
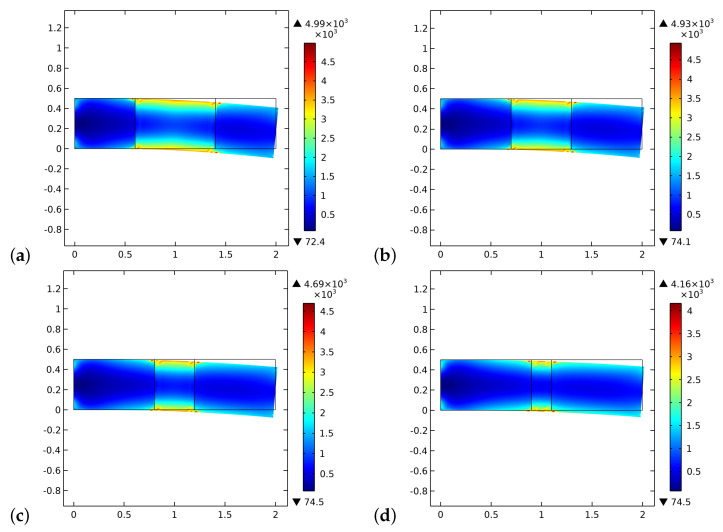
The distribution of the energy density E at the end of the time of simulation for different substitute lengths of the graft: (**a**) 0.8 cm. (**b**) 0.6 cm. (**c**) 0.4 cm. (**d**) 0.2 cm.

Figure 8 and Figure 9 exhibit the initial and final distributions of elastic energy density E. The system becomes stiffer since the stored energy decreases during the process. The external actions clearly induce bending of the sample with a localization of the energy close to the longest verges. The effect of the interface is less evident than that of the ζ field.

**Figure 10:**
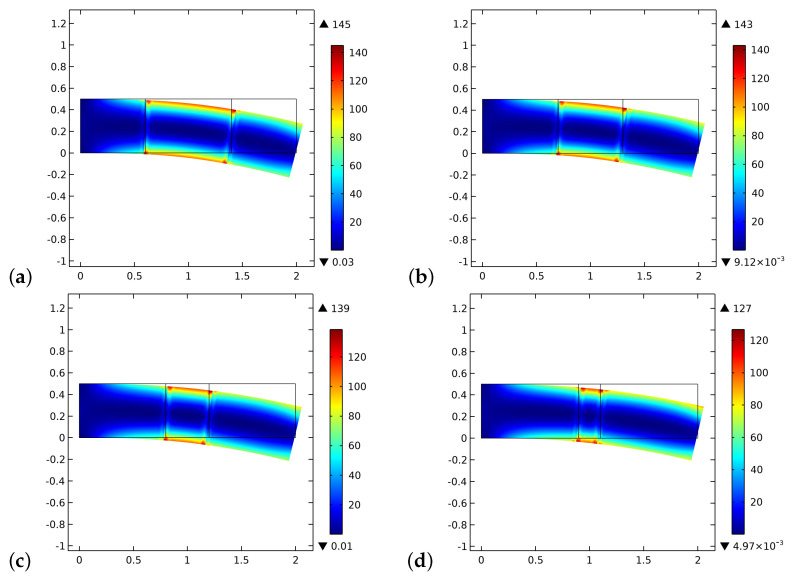
The distribution of the dissipated energy density at the beginning of the time of simulation for different substitute lengths of the graft: (**a**) 0.8 cm. (**b**) 0.6 cm. (**c**) 0.4 cm. (**d**) 0.2 cm.

**Figure 11:**
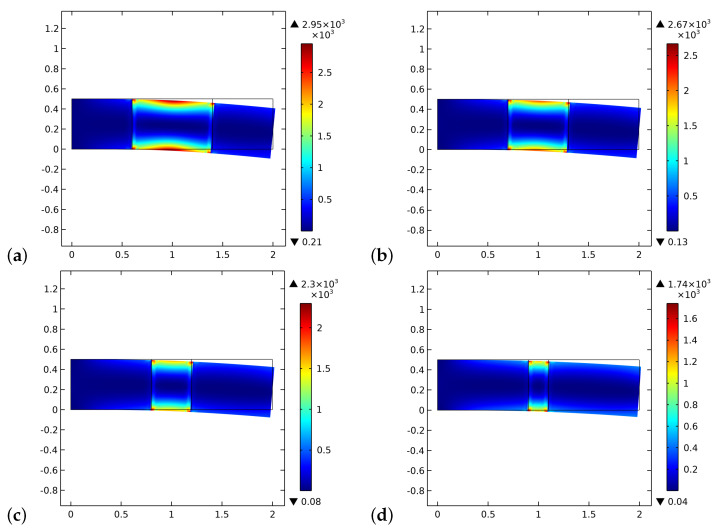
The distribution of the dissipated energy density at the end of the time of simulation for different substitute lengths of the graft: (**a**) 0.8 cm. (**b**) 0.6 cm. (**c**) 0.4 cm. (**d**) 0.2 cm.

Figure 10 and Figure 11 plot the initial and final distributions of the dissipated energy density. On the contrary of the elastic energy density, the dissipated energy density increases as the simulation progresses. We also note a different distribution of it, particularly a significant localization all along the boundaries of the graft.

The numerical simulations show that for externally applied loads sufficiently high, the graft can be partially substituted by bone tissue. The composition of the final bone/graft composite in the graft zone primarily depends not only on the intensity of the mechanical load, which is a source of the stimulus, but also on the rates of bone synthesis and resorption of both phases and the size of the graft. As a matter of fact, the larger is the graft, the more resorption may occur far from the bone tissue where no osteocytes are present. This increase in porosity facilitates new bone production in the remodeling process that substitutes the graft material. Besides, the stimulus is nonlocal in nature [30], and therefore if the graft is sufficiently small, the influence due to the near living bone compensates for the absence of osteocytes within the graft at the beginning of the process. Thus, for small grafts, the synthesis of bone can start even before without or with a small extent of artificial material resorption. This qualitative behavior is in accordance with real bone healing cases, for which the process is favored for small gaps rather than large ones. In these cases, the nonlocal stimulus from the not damaged tissue triggers the healing faster and starts on the proximity of the healing process from which the stimulus originates. These results align with those of other works with similar formulations present in the literature (see, e.g., [35,40]).

We remark that the formulated model is based on the hypothesis that after the reconstruction surgery through the graft, this last is flood over by blood vessels, lymphatic vessels, nerves, and osteoblasts and osteoclasts responsible for the evolution of the system. After this stage, we have begun the simulations of the bone/graft evolution. To have a more detailed comprehension of the entire process, a proper description of the starting stage of the healing process is still missing in the proposed formulation. Therefore, this aspect is left open for further investigations.

## 5. Conclusions

This paper aims to show that with a relatively coarse model (without the details of the microstructure characterizing the system), it is possible to analyze the macroscopic bio-mechanical behavior of a system constituted by bone and artificial bio-resorbable material with decent accuracy. Therefore, after a brief introduction of the model employed, some numerical simulations are performed to confirm that the proposed formulation is qualitatively able to provide aid in the design of the graft to be used in bone reconstruction surgery. Naturally, the model parameters must be calibrated with experimental data, but this is always an obliged step. Assuming for the graft a mechanical micro-structure similar to that of the bone, we have shown that its size greatly influences the aptitude of the graft to be substituted with the newly synthesized bone. With this example in mind, we believe that the proposed model is a powerful tool for efficiently engineering the graft in an initial design phase by optimizing its macroscopic characteristics, which clearly requires many numerical simulations.

## Figures and Tables

**Table 1:** Material parameters used in the numerical simulations.

$Y_{b}^{\mathrm{Max}}$ (GPa)	$Y_{m}^{\mathrm{Max}}$ (GPa)	ν	${\hat{\rho }}_{b}$ (kg/m^3^)	${\hat{\rho }}_{m}$ (kg/m^3^)
17	13.6	0.3	1800	1800
$K_{f}$ (GPa)	$K_{2}$ (N)	$K_{u}$ (N/m^3^)	$K_{\nabla u}$ (N/m)	$K_{\zeta }$ (N/m)
1.7	$1.7\times 10^{5}$	$1.7\times 10^{18}$	$1.7\times 10^{7}$	$1.7\times 10^{7}$
$\kappa^{v}$ (N s/m^2^)	$\mu^{v}$ (N s/m^2^)	$s_{b}$ (s/m^2^)	$r_{b}$ (s/m^2^)	$r_{m}$ (s/m^2^)
$2.06\times 10^{12}$	$2.57\times 10^{12}$	$1.27\times 10^{-7}$	$1.06\times 10^{-7}$	$1.59\times 10^{-7}$
*D* (mm)	$a_{1}$	$a_{2}$	$P_{\mathrm{ref}}^{r}$ (N/m^2^)	$P_{\mathrm{ref}}^{s}$ (N/m^2^)
1	1	1	50.97	56.33

## Data Availability

Not applicable.

## Abbreviations

The following abbreviations are used in this manuscript:

RMS	root mean square
PD	proportional derivative
BGB	bone-graft-bone
